# Comparative effects of enzymatic soybean, fish meal and milk powder in diets on growth performance, immunological parameters, SCFAs production and gut microbiome of weaned piglets

**DOI:** 10.1186/s40104-021-00625-8

**Published:** 2021-10-07

**Authors:** Yingjie Li, Yang Liu, Jiangnan Wu, Qiuhong Chen, Qiang Zhou, Fali Wu, Ruinan Zhang, Zhengfeng Fang, Yan Lin, Shengyu Xu, Bin Feng, Yong Zhuo, De Wu, Lianqiang Che

**Affiliations:** Key Laboratory for Animal Disease-Resistance Nutrition of China Ministry of Education, Institute of Animal Nutrition, Sichuan Agricultural University, No. 211, Huimin Road, Wenjiang District, Chengdu, 611130 Sichuan People’s Republic of China

**Keywords:** Enzymatic soybean, Growth performance, Gut microbiome, Immunology, SCFAs, Weaned piglets

## Abstract

**Background:**

The objective of this study was to evaluate the replacement effects of milk powder (MK) and fish meal (FM) by enzymatic soybean (ESB) in diets on growth performance, immunological parameters, SCFAs production and gut microbiome of weaned piglets.

**Methods:**

A total of 128 piglets with initial body weight at 6.95 ± 0.46 kg, were randomly assigned into 4 dietary treatments with 8 replicates per treatment and 4 piglets per replicate for a period of 14 d. Piglets were offered iso-nitrogenous and iso-energetic diets as follows: CON diet with MK and FM as high quality protein sources, ESB plus FM diet with ESB replacing MK, ESB plus MK diet with ESB replacing FM, and ESB diet with ESB replacing both MK and FM.

**Results:**

No significant differences were observed in growth performance among all treatments (*P >* 0.05). However, piglets fed ESB plus FM or ESB diet had increased diarrhea index (*P*<0.01), and lower digestibility of dry matter (DM), gross energy (GE) or crude protein (CP), relative to piglets fed CON diet (*P* <  0.01). Moreover, the inclusion of ESB in diet markedly decreased the plasma concentration of HPT and fecal concentration of butyric acid (BA) (*P*<0.01). The High-throughput sequencing of 16S rRNA gene V3−V4 region of gut microbiome revealed that the inclusion of ESB in diet increased the alpha diversity, and the linear discriminant analysis effect size (LEfSe) showed that piglets fed with ESB plus FM or ESB diet contained more gut pathogenic bacteria, such as *g_Peptococcus*, *g_Veillonella* and *g_Helicobacter*.

**Conclusion:**

The inclusion of ESB in diet did not markedly affect growth performance of piglets, but the replacement of MK or both MK and FM by ESB increased diarrhea index, which could be associated with lower nutrients digestibility and more gut pathogenic bacteria. However, piglets fed diet using ESB to replace FM did not markedly affect gut health-related parameters, indicating the potential for replacing FM with ESB in weaning diet.

## Background

Weaning is a critical period for the growth and development of piglets, during which weaned piglets have to cope with a series of problems such as dysfunction of intestinal barrier and systematic inflammation induced by weaning stress, thereby aggravate diarrhea, morbidity and mortality, and poor growth performance [1]. During the suckling period, piglets ingest high digestible milk of sows as the major food. However, the newly weaned piglets are abruptly forced to adapt to the nutritional and environmental changes, especially digest solid diets containing high contents of plant proteins with the immature digestive and immune systems, which has been demonstrated to aggravate the weaning stress [2, 3]. Thus, it is extremely urgent to incorporate the high quality protein sources into diet to prevent weaning stress in piglets [4].

Fish meal (FM) and milk powder (MK) are extensively used in creep feed as high quality protein sources due to the higher digestibility, greater palatability and appropriate composition of amino acids [5, 6]. However, the exorbitant cost of FM and MK have necessitated the identification of alternative cheaper protein sources for the weaning diets [7, 8]. Although soybean has become the primary protein source in swine diet due to the excellent balance of essential amino acids and lower price [9], soybean is not recommendable to be directly used in weaning diets due to its anti-nutritional factors (ANFs), which can cause hypersensitivity [10–12].

It is confirmed that bioprocessing of soybean is an effective way to eliminate ANFs and improve the bioavailability of diet [13, 14]. The enzymatic soybean (ESB), produced by fermentation and enzymatic hydrolysis of soybean, is an excellent protein source with less trypsin inhibitors and antigen proteins [15, 16]. In addition, It has been reported that growth performance, antioxidant capacity, immune function and nutrients digestibility of weaned piglets could be improved as the ESB was incorporated into diet to replace some other dietary protein sources such as soybean meal (SBM), soybean protein concentrate (SPC), fermented soybean meal (FSBM) or FM [17–19]. However, to the best of our knowledge, there is short of researches regarding the comparison of employing ESB to completely substitute for FM, MK or both FM and MK in weaning diets. Hence, the objective of the study was to assess the comparative effects of FM, MK or both MK and FM replacing with ESB on growth performance, nutrients digestibility, immunological parameters, gut microbiome and short-chain fatty acids (SCFAs) in weaned piglets.

## Materials and methods

The experiment followed the animal protection law (Ethic Approval Code: SCAUAC201308–2) and was performed in accordance with the Guide for the Animal Care and Use approved by Sichuan Agricultural University Institutional Animal Care and Use Committee.

### Preparation of ingredients

The MK was obtained from Fonterra, New Zealand. The FM was produced by Pesquera diamante, Peru. The ESB, which was obtained from Fatide, Jiangsu Fuhai Biology Co., Ltd., contained 40.00% crude protein (CP), 18.00% fat, 2.80% fiber, and lower ANFs (0.13% stachyose, 0.39% raffinose, 126.32 TIU/g trypsin inhibitor, β-conglycinin and glycinin are less than 1.4 mg/g and 2.8 mg/g respectively) compared with unprocessed soybean.

### Animals, diets, and experimental design

A total of 128 piglets ((Landrace × Yorkshire × Duroc) × Yorkshire; 21d ± 2d) with an initial body weight at 6.95 ± 0.46 kg were randomly assigned into 4 dietary treatments in a randomized complete block design according to body weight: (1) CON diet with MK and FM as high quality protein sources; (2) ESB plus FM diet with ESB replacing MK; (3) ESB plus MK diet with ESB replacing FM; (4) ESB diet with ESB replacing both MK and FM. Each treatment group had 32 piglets with 8 pens and 4 piglets (2 barrows and 2 gilts) per pen for the 14-d experiment. As presented in Table 1, all the diets were formulated to be iso-nitrogenous and iso-energetic and meet or exceed the recommendation of NRC (2012) [20]. Piglets were housed in pens (1.5 m × 1.5 m) with infrared lamps hanged above and the temperature was kept between 26 and 28 °C. Piglets had free access to feed and water during the experimental period.

**Table 1:** The ingredient composition and analyzed nutrient levels of diets (as fed basis)

	CON	ESB Plus FM	ESB plus MK	ESB
Ingredient, %				
Corn	44.98	43.57	42.28	40.82
Soybean concentrate protein	4.00	4.00	4.00	4.00
Dehulled soybean meal	4.50	4.50	4.50	4.50
Fermented soybean meal	4.00	4.00	4.00	4.00
Extruded soybean	4.00	4.00	4.00	4.00
Whey powder	15.00	15.00	15.00	15.00
Enzymatic soybean	–	8.50	6.70	15.20
Milk powder	10.00	–	10.00	–
Fish meal	4.00	4.00	–	–
Sucrose	5.00	5.00	5.00	5.00
Soybean oil	1.30	3.40	0.50	2.60
L-Lysine·HCl	0.49	0.60	0.49	0.61
DL-Methionine	0.26	0.32	0.30	0.36
L-Threonine	0.23	0.23	0.23	0.24
L-Tryptophan	0.06	0.07	0.05	0.06
L-Valine	0.05	0.13	0.05	0.13
Choline chloride	0.16	0.16	0.16	0.16
Calcium formate	0.80	0.90	1.12	1.20
CaHPO4	0.10	0.45	0.55	0.90
NaCl	–	0.10	–	0.15
Mineral premix^a^	0.20	0.20	0.20	0.20
Vitamin premix^b^	0.05	0.05	0.05	0.05
ZnO, 65%	0.20	0.20	0.20	0.20
Emulsifier	0.10	0.10	0.10	0.10
Benzoic acid	0.50	0.50	0.50	0.50
Essential oils	0.02	0.02	0.02	0.02
Total	100.00	100.00	100.00	100.00
Calculated nutrient levels				
DE, MJ/kg	15.09	15.09	15.08	15.08
CP, %	19.28	19.44	19.12	19.29
Lys, %	1.50	1.50	1.49	1.50
Met, %	0.62	0.63	0.63	0.63
Thr, %	0.93	0.93	0.93	0.93
Analyzed nutrient levels				
GE, MJ/kg	18.24	18.47	18.21	18.56
CP, %	19.22	20.43	19.23	20.27
DM, %	98.62	95.61	96.70	94.91
Total AA, %	18.41	18.02	18.40	18.17
Lys, %	1.53	1.53	1.56	1.53
Met, %	0.53	0.56	0.54	0.53
Thr, %	1.33	1.17	1.05	1.37

*GE*, gross energy

^a^ Mineral premix provided per kilogram of diet: Fe, 100 mg; Cu, 6 mg; Zn, 100 mg; Mn, 4 mg; I, 0.14 mg; Se, 0.35 mg

^b^ Vitamin premixes provided per kilogram of diet: vitamin A, 15,000 IU; vitamin D_3_, 5000 IU; vitamin E, 40 mg; vitamin K_3_ 5 mg; vitamin B_1_ 5 mg; vitamin B_2_ 12.5 mg; vitamin B_6_ 6 mg; vitamin B_12_ 0.06 mg; nicotinic acid, 50 mg; pantothenic acid, 25 mg; folic acid, 2.5 mg; biotin, 0.25 mg

### Growth performance

The feed supply and feed refusals were recorded every day, and piglets were individually weighted every week to calculate average daily gain (ADG), average daily feed intake (ADFI) and the ratio of ADFI and ADG (F:G). Diarrhea scores were visually assessed three times a day as previous described [21]. Briefly, firm and well-formed feces were scored as 0; soft and formed feces were scored as 1; fluid and usually yellowish feces were scored as 2; and watery and projectile feces were scored as 3. Diarrhea index was calculated according to the following equation: diarrhea index = the sum of diarrhea scores / (numbers of piglets per pen × experimental days × assessed times per day).

### Sample collection

Blood samples (10 mL, *n* = 8) were collected from jugular vein into sodium heparinized tubes at 08:00 of d 8 after an overnight fast. Plasma was obtained by centrifuging at 3000 × *g* for 15 min at 4 °C and stored immediately at − 20 °C for later analysis. At the same day, fresh fecal samples (*n* = 8) were collected by rectal stimulation, then snap frozen at − 80 °C for the gut microbiome analysis.

To determine the nutrients digestibility of piglets, 0.5% chromic oxide was additional added to the diets as an exogenous indicator on d 8. After 4-d adaptation period, fresh fecal samples were collected during d 12 to d 14. The diet and fecal samples for nutrients digestibility determination were stored at − 20 °C until analysis.

### Chemical analysis

The diets and feces samples were dried at 65 °C for 72 h, ground through a 0.42-mm screen and analyzed according to methods of AOAC for dry matter (DM) [22]. CP was determined by copper catalyst Kjeldahl method and GE was determined by an automatic adiabatic oxygen bomb calorimeter (Parr 6400, Parr Instrument Co., Moline, IL, USA). Amino acids, except tryptophan, were measured by an automatic amino acid analyzer (L-8900, Hitachi, Tokyo, Japan) after acidolysis for 24 h. Chromium was determined by a flame atomic absorption spectrophotometer (ContrAA 700, Analytikjena, Jena, Germany). The Apparent total tract digestibility (ATTD) was calculated according to the following equation: ATTD_nutrient_ = 1 − (Cr_diet_ × Nutrient_feces_) / (Cr_feces_ × Nutrient_diet_) [23].

### Measurement of plasma parameters

Plasma samples were thawed on the ice before analysis. The 300 μL of supernatant were obtained to determine the concentrations of plasma immunoglobulin A (IgA) and immunoglobulin G (IgG) via automatic biochemical analyzer (Hitachi 3100, Hitachi High-Technologies Corporation, Tokyo, Japan) with corresponding kits (Sichuan Maker Biotechnology Co. Ltd). The levels of haptoglobin (HPT) and pig major acute-phase protein (Pig-MAP) were measured by spectrophotometric methods (Spectra Max M2; Molecular Devices, California, USA), according to the kit instructions (Nanjing Jiancheng Bioengineering Institute, Nanjing, China). There was less than 5% variation of intra-assay and inter-assay coeffcients for each assay.

### Quantification of SCFAs

The concentrations of SCFAs were determined by gas chromatography (Varian CP-3800, manual injection, flame ionization detector, FID, 10 μL micro-injector). Approximately 0.7 g of fecal samples were thawed and diluted with 1.5 mL of ultrapure water, and 1.0 mL supernatant was obtained by centrifuging at 3000 × *g* for 15 min. Then the supernatant was mixed with 0.2 mL of 25% metaphosphoric acid solution and 23.3 μL of 210 mmol/L crotonic acid and the mixed solution was placed at 4 °C for 30 min before centrifuging at 4000 × *g* for 10 min, afterwards the 0.3 mL of supernatant was mixed with 0.9 mL of methanol, filtered by 0.22-μm filter (Millipore Co., Bedford, MA) after centrifuging at 3500 × *g* for 5 min.

### Sequencing of gut microbiome

The total genomic DNA was extracted from fecal samples (*n* = 8) using the QIAamp DNA stool Mini Kit (Qiagen, GmbH Hilden, Germany). The concentration and purity of the extracted genomic DNA were measured using a NanoDrop ND-1000 Spectrophotometer (NanoDrop Technologies Inc., Wilmington, DE, USA). The integrity of the extracted genomic DNA was determined by electrophoresis on 1% (w/v) agarose gels. Extracted fecal DNA samples were sent to Majorbio Bio-pharm Technology Co., Ltd. (Shanghai, China) to perform amplicon pyrosequencing on the Illumina MiSeq platforms. The V3−V4 hypervariable region of the 16S rRNA gene was amplified by PCR with primers 338F (5′-ACTCCTACGGGAGGCAGCAG-3′) and 806R (5′-GGACTACHVGGTWTCTAAT-3′). The Uparse 7.0.1090 was used to clustered Operational taxonomic units (OTUs) at 97% sequence similarity. Representative sequences were selected and assigned by the Ribosomal Database Project (RDP) classifier Version 2.11. The relative abundance of each OTU was examined at different taxonomic levels. Diversity within communities (Alpha diversity) calculations and taxonomic community assessments were performed by Mothur 1.30.2 and Qiime 1.9.1. Principal coordinates analysis (PCoA) plots were produced using unweighted UniFrac metrics. The linear discriminant analysis (LDA) effect size (LEfSe) method was performed to elucidate the difference among treatments.

### Statistical analysis

Data were analyzed using PROC MIXED of SAS 9.4 (SAS Inst. Inc., Cary, NC, USA). The data were considered as outlier when the student residue was greater than three. The UNIVARIATE procedures of SAS were used to analyze the variance homogeneity and normality, respectively. Least squares means were calculated using the LSMEANS procedure in SAS, and significant differences among treatments were separated using PDIFF option with the Tukey adjustment for the performance data. For growth performance, diarrhea index, ATTD and SCFAs, pens were regarded as the experimental unit and piglet data were reported as a mean for the pen. The different taxa microbes among lines were identified using LEfSe analysis with LDA score > 3. Data are presented as the least squares means and pooled standard error. Results were considered significant when *P* < 0.05 and tendency toward significance at 0.05 ≤ *P* < 0.10.

## Results

### Growth performance

As presented in Table 2, piglets fed CON or ESB plus MK diet had lower diarrhea index (*P* <  0.01) during d 1–7 and the whole experimental period, relative to piglets fed ESB plus FM or ESB diet. Besides, piglets fed CON or ESB plus MK diet had lower diarrhea index (*P* <  0.01) during d 8–14, when compared with piglets fed ESB diet. There was no significant difference among dietary treatments in ADG, ADFI and F:G at all phase.

**Table 2:** Effects of dietary protein sources on growth performance and diarrhea index in weaned piglets

	CON	ESB plus FM	ESB plus MK	ESB	SEM	*P*-value
Body Weights, kg						
d 1	6.95	6.94	6.96	6.95	0.02	0.85
d 7	8.00	7.84	8.00	7.93	0.44	0.34
d 14	10.35	9.84	10.01	9.92	0.69	0.14
d 1–7						
ADG, g/d	149	128	148	138	23	0.48
ADFI, g/d	275	260	252	280	45	0.76
F:G	1.95	2.07	1.77	2.05	0.36	0.51
Diarrhea index	0.08^b^	0.13^a^	0.08^b^	0.13^a^	0.02	< 0.01
d 8–14						
ADG, g/d	337	287	288	286	51	0.20
ADFI, g/d	503	482	480	483	65	0.91
F:G	1.50	1.73	1.71	1.77	0.20	0.13
Diarrhea index	0.11^b^	0.17^ab^	0.10^b^	0.24^a^	0.03	< 0.01
d 1–14						
ADG, g/d	243	207	218	211	31	0.19
ADFI, g/d	389	371	366	387	48	0.82
F:G	1.62	1.80	1.71	1.87	0.17	0.14
Diarrhea index	0.14^b^	0.22^a^	0.14^b^	0.25^a^	0.02	< 0.01

^a-b^ Mean values within a row with different letters differ at *P* < 0.05

### The ATTD of nutrients

As presented in Table 3, the digestibility of DM, CP and GE were significantly lower in the piglets fed ESB plus FM diet (*P* < 0.01), when compared with piglets fed CON diet. The DM, CP and GE digestibility did not markedly differ between piglets fed ESB plus MK diet and CON diet (*P* > 0.05), but piglets fed ESB plus MK diet had significantly higher digestibility of DM and GE than that of ESB plus FM group (*P* <  0.01). Besides, piglets fed ESB diet had markedly decreased digestibility of DM and GE, relative to piglets fed CON diet (*P* <  0.01).

**Table 3:** Effects of dietary protein sources on ATTD of nutrients in weaned piglets

Items, %	CON	ESB plus FM	ESB plus MK	ESB	SEM	*P*-value
DM	88.54^a^	84.45^c^	87.62^ab^	86.63^b^	0.92	< 0.01
CP	83.70^a^	78.02^b^	81.10^ab^	81.46^a^	1.71	< 0.01
GE	88.30^a^	83.53^c^	87.14^ab^	85.71^b^	1.11	< 0.01

^a-c^ Mean values within a row with different letters differ at *P* < 0.05

### Immunological parameters

**Fig. 1:**
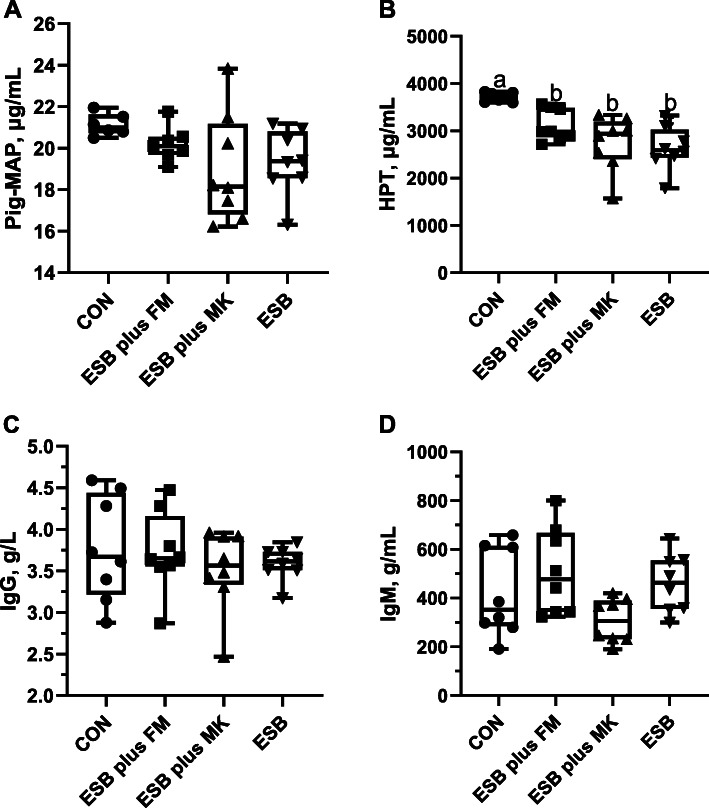
Effects of dietary protein sources on immunological parameters in weaned piglets

As presented in Fig. 1, the plasma concentrations of Pig-MAP, IgG and IgM were not markedly affected by dietary treatments (*P* > 0.05). Piglets fed ESB plus FM, ESB plus MK and ESB had markedly decreased plasma concentration of HPT when compared with piglets fed CON diet (*P* <  0.01).

### Gut microbiome

**Fig. 2:**
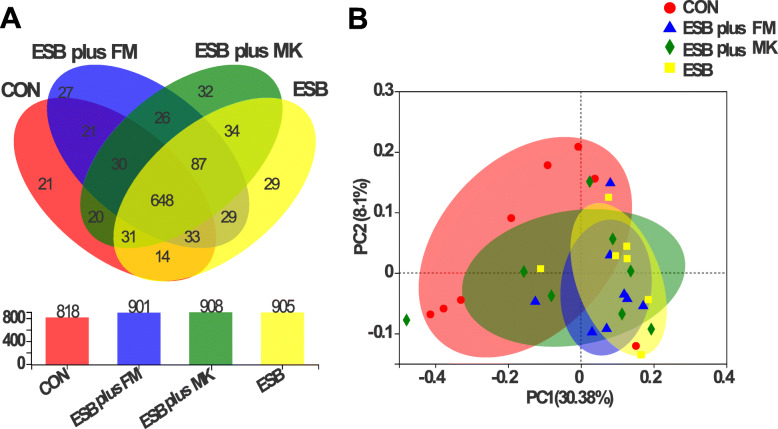
Effects of dietary protein sources on gut microbiome structure in weaned piglets. Venn diagram showing the unique and shared OTUs in the different groups (**A**). Unweighted Unifrac PCoA analysis based on OTUs of gut microbiome (**B**)

A total of 41,562, 46,965, 53,874 and 50,331 effective sequences in fecal samples from CON, ESB plus FM, ESB plus MK and ESB groups were identified, respectively. From the Venn analysis of OTUs, 818, 901, 908 and 905 unique OTUs were identified in CON, ESB plus FM, ESB plus MK and ESB groups, respectively (Fig. 2A). For beta diversity analysis, unweighted Unifrac PCoA was performed to demonstrate the separation of bacterial community composition among treatments by using the first two principal component scores of PC1 and PC2 (30.38% and 8.1%) of the explained variance, respectively (Fig. 2B).

As shown in Table 4, dietary treatments did not markedly affect Shannon, Simpson and ACE indexes, but piglets fed ESB or ESB plus FM diets had significantly increased Chao 1 index when compared with piglets fed CON diet (*P* <  0.05).

**Table 4:** The alpha diversity in the fecal microbiome of weaned piglets

	CON	ESB plus FM	ESB plus MK	ESB	SEM	*P*-value
Shannon	0.98	1.05	0.96	1.04	0.11	0.54
Simpson	0.44	0.43	0.43	0.43	0.09	0.99
ACE	10.51	13.07	12.27	15.80	0.21	0.11
Chao 1	9.56^b^	12.56^a^	11.81^ab^	14.06^a^	2.01	0.03

^a-b^ Mean values within a row with different letters differ at *P* < 0.05

**Fig. 3:**
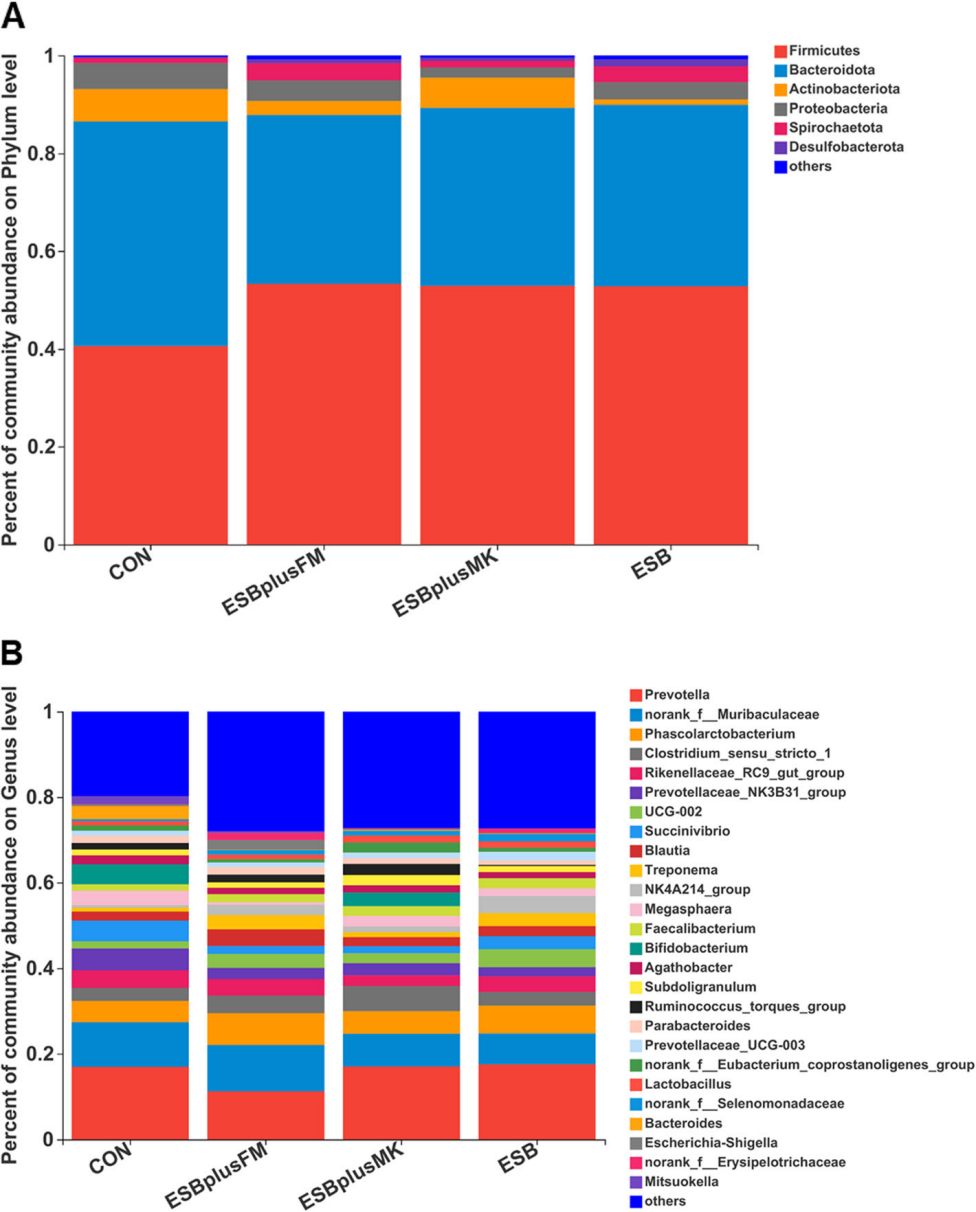
Effects of dietary protein sources on phyla and genus of gut microbiome in weaned piglets. Relative abundances of phyla (**A**) and genus (**B**). The abundance is expressed in terms of a percentage of the total effective bacterial sequences in the fecal samples

The relative abundances at phylum level among treatments are presented in Fig. 3A, suggesting that the top 6 dominated phyla were Firmicutes, Bacteroidota, Actinobacteriota, Proteobacteria, Spirochaetota and Desulfobacterota. Firmicutes occupied the maximal portion of gut microbiome in all samples, with a relative abundance of 50%. At the genus level, a total of 248 genera were identified among all samples, and the top 26 genera (> 1.5% in at least one group) are shown in Fig. 3B.

**Fig. 4:**
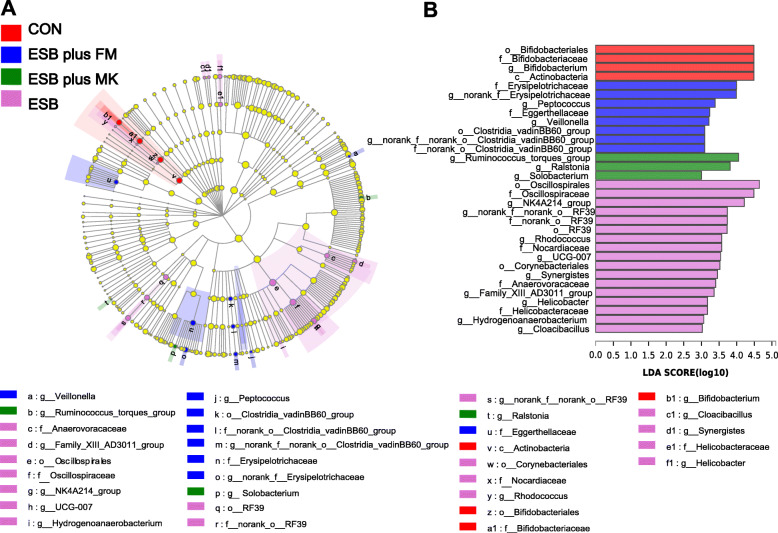
Effects of dietary protein sources on the phylotypes distribution in weaned piglets. LefSe cladogram representing differential abundant taxa in pig gut microbiome (**A**). LefSe bar representing differential abundant taxa in pig gut microbiome (**B**)

LEfSe was used to analyze microbial community from phylum to genus level. There were 4, 8, 3 and 17 kinds of dominant bacteria in fecal samples of piglets fed CON, ESB plus FM, ESB plus MK and ESB diets respectively (Fig. 4). The most abundant phylotypes in fecal samples of piglets fed ESB plus FM diet were o_Clostridia_vadinBB60_group, f_Erysipelotrichaceae, *g_Peptococcus*, and *g_Veillonella*. And o_RF39, f_Oscillospiraceae, f_Nocardiaceae and *g_Helicobacter* were enriched in fecal samples of piglets fed ESB diet.

### Concentrations of SCFAs in feces

The results pertaining to the SCFAs in feces are presented in Table 5. Compared with piglets fed ESB plus FM, ESB plus MK and ESB diets, piglets fed CON diet had significantly increased level of butyric acid (BA) in feces (*P* <  0.01) and tended to have higher level of acetic acid (AA) (*P* = 0.09). The level of propionic acid (PA) in feces was not significant different across the dietary treatments (*P *> 0.05).

**Table 5:** Effects of dietary protein sources on the SCFAs levels in fecal samples in weaned piglets

Items, μmol/mg	CON	ESB plus FM	ESB plus MK	ESB	SEM	*P*-value
AA	6.63	5.00	6.16	4.37	0.56	0.09
PA	1.84	1.29	1.53	1.35	0.38	0.20
BA	4.24^a^	2.05^b^	1.76^b^	1.13^b^	0.67	<0.01

^a-b^ Mean values within a row with different letters differ at *P* < 0.05

## Discussion

Soybean is commonly used in the diets of pigs because of its high content of proteins and greater digestibility [24, 25]. However, ANFs in soybean may have negative impact on the immature gastrointestinal tract of weaned piglets, which may result in severe diarrhea [26]. Bio-processed soybean products, such as SPC, FSBM and ESB, have been demonstrated to remove ANFs effectively and improve nutrients digestibility, which leads to a better growth performance in weaned piglets [27, 28]. In addition to decrease ANFs, the fermentation and enzymatic hydrolysis process of ESB also produces more small peptides, which has various physiological function in piglets or other mammals [29–31].

In the present study, the growth performance of weaned piglets did not markedly differ among treatments, however, we did observe the growth performance of piglets fed ESB plus FM or ESB diet had been numerically decreased, as indicated by the average 14% decrease in whole period ADG.

Protein digestion has been proposed to be a major dietary factor affecting growth and diarrhea incidence of weaned piglets [32], as the undigested dietary protein enters into the hindgut leading to altered gut microbiome [33]. In this study, we observed the diarrhea index was markedly increased by feeding ESB plus FM diet or ESB diet, which could be partially ascribed to the poor nutrient digestibility.

For weaning piglets, weaning stress is a vital factor that causing immunological and intestinal impairments [34]. The immunoglobulins and acute phase proteins, such as IgG, IgM, Pig-MAP and HPT, could regulate immunity by inhibiting the release of IL-1 and TNF-α [35, 36]. As an inflammatory signaling factor, the plasma HPT concentration could be particularly elevated during the occurrence of inflammation and injury [37]. Our study showed that piglets fed ESB plus FM, ESB plus MK and ESB diets had dramatically decreased plasma concentration of HPT, which is consistent with previous study that piglets fed enzymolytic soybean meal had improved immune function, as indicated by the higher levels of CD4+ and CD8+ in peripheral blood [38]. The immunoregulatory effect of bio-processed soybean may be related to the functional bio-active peptides. Supportively, the small peptides account for up to 33.58% of total protein in ESB we used in this study. Similarly, recent studies have reported that the size of soybean peptide could be reduced to 100 ~ 1000 Da by microbial fermentation and proteolysis during the production of ESB, containing abundant bio-active peptides, such as QRPR and lunasin [39–41].

Gut microbiome has been shown to play an important role in development and function of weaned piglets [42]. In our study, the 16S rRNA sequencing was used to investigate the gut microbiome responses to dietary protein sources in weaned piglets. Our results showed that the piglets fed ESB plus MK diet contained the most OTUs. Besides, piglets fed ESB plus FM diet or ESB diet increased Chao 1 index (*P* = 0.03), and piglets fed ESB diets increased ACE index by 44% ~ 67%, indicating that the inclusion of ESB in diet increased gut microbial richness and diversity in weaned piglets .

To elucidate the difference in microbiome among treatments, LefSe method was conducted to analyze the enriched bacteria in each group. In the present study, piglets fed ESB plus FM or ESB diet had increased the abundances of some pathogenic bacteria in feces, such as o_Clostridia_vadinBB60_group, o_RF39, f_Erysipelotrichaceae, f_Oscillospiraceae, f_Nocardiaceae, *g_Peptococcus*, *g_Veillonella* and *g_Helicobacter.* It has been reported that o_Clostridia_vadinBB60_group and f_Erysipelotrichaceae were enriched in the lumen of colorectal cancer patients [43, 44]. In addition, g*_Peptococcus* is a classic pathogenic bacteria colonized in animals with gastrointestinal disease, and *g_Veillonella* was negatively correlated with the nutritional index [45, 46]. The higher abundances of o_Oscillospiraceae and *g_Helicobacter* have been found to be related to intestinal inflammation [47, 48]. The o_RF39, which belongs to phylum Tenericutes, class Mollicutes, is associated with intestinal disorders [49, 50]. Furthermore, f_Nocardiaceae belongs to phylum Actinobacteria, is a strong predictor of diarrhea in piglets [51]. Taken together, the significantly higher diarrhea index in piglets fed ESB plus FM or ESB diet may be attributed to the intestinal damage induced by the enriched pathogenic bacteria.

SCFAs, the main metabolites produced by bacterial fermentation of dietary fiber and protein in the large intestine, can regulate the absorption of various nutrients and provide nearly 30% of the energy requirements for maintenance in pigs and then improve piglets performance [52–54]. In the current study, we found that piglets fed CON diet had higher abundance of *g_Bifidobacterium* in feces, which has been demonstrated to suppress the colonization of pathogenic bacteria and contribute to the production of SCFAs [55–57]. Supportively, we did observe the increased AA and BA levels in piglets fed CON diet.

## Conclusion

In this study, the inclusion of ESB in weaning diet did not markedly affect growth performance of piglets, but the substitution of MK or both MK and FM with ESB in diet leaded to higher diarrhea index, which could be ascribed to the lower nutrients digestibility and more gut pathogenic bacteria, such as *g_Veillonella*, *g_Helicobacter* and g*_Peptococcus*. However, piglets fed diet using ESB to replace FM did not markedly affect gut health-related parameters, indicating the potential for replacing FM with ESB in weaning diet.

## Data Availability

The datasets analyzed in the current study are available from the corresponding author on reasonable request.

## Abbreviations

MK: Milk powder
FM: Fish meal
ESB: Enzymatic soybean
SCFAs: Short-chain fatty acids
DM: Dry matter
CP: Crude protein
GE: Gross energy
LEfSe: Linear discriminant analysis effect size
LDA: Linear discriminant analysis
ANFs: Anti-nutritional factors
ADG: Average daily gain
ADFI: Average daily feed intake
F:G: Feed to gain ratio
ATTD: Apparent total tract digestibility
HPT: Haptoglobin
Pig-MAP: Pig major acute-phase protein
OTUs: Operational taxonomic units
RDP: Ribosomal Database Project
PCoA: Principal coordinates analysis
AA: Acetic acid
PA: Propionic acid
BA: Butyric acid
